# Plant Food, Nutrition, and Human Health

**DOI:** 10.3390/nu12072157

**Published:** 2020-07-20

**Authors:** Pedro Mena, Donato Angelino

**Affiliations:** 1 Department of Food and Drug, Human Nutrition Unit, University of Parma, 43125 Parma, Italy; 2 Faculty of Bioscience and Technology for Food, Agriculture, and Environment, University of Teramo, 64100 Teramo, Italy

Phytochemical compounds are non-nutrient secondary metabolites present in plant-based foods, e.g., fruits, vegetables, whole grains, legumes. Among the different classes of phytochemicals, phenolic compounds, glucosinolates, and other sulfur compounds, carotenoids, alkaloids, and terpenes have been widely studied for their beneficial effects on human health. For this reason, they are also called “bioactive compounds”, as their intake seems to impact different biological processes. However, the elucidation of their metabolic fate and bioavailability is a tipping point to fully unravel the preventive effects of plant bioactives on chronic diseases, including among others, cardiovascular diseases, metabolic syndrome, neurodegenerative disorders, and certain kinds of cancer. In this scenario, gut microbiota, and, on the whole, the inter-person variability have been shown to impact the chemical structure and in vivo metabolism of phytochemicals at an almost individual level. These facts point out that not only parent compounds, but also circulating metabolites, might have a role in exerting biological activity, and that the “one-size-fits-all approach” does not necessarily explain the biological response to the consumption of plant bioactives. This Special Issue provided a series of 23 original contributions, among which 17 research articles and six reviews, dealing with some of the multiple aspects behind the intake, metabolism, and biological activity of glucosinolates, carotenoids, and (poly)phenols, as well as their circulating metabolites. 

Among these papers, some focused on plants for which there is a growing interest in elucidating the potential effects of their bioactive compounds on different outcomes. For instance, *Moringa oleifera* (MO), that is a cruciferous plant belonging to the *Moringaceae* family, has been particularly taken into consideration in this Special Issue, with three original studies and a narrative review. In this latter work, Kou et al. [1] summarized the recent evidence from in vitro and in vivo studies on anti-inflammatory and antioxidant activities, as well as the effects on cognitive and cancer-related outcomes of compounds—mainly glucosinolates and isothiocyanates—coming from MO seeds, stem bark, leaves, or root bark. MO is well known for the high content of a benzyl glucosinolate–glucomoringin, which once being in contact with myrosinase enzyme, results in the production of the benzyl isothiocyanate, moringin. Fahey et al. [2] demonstrated the effective biological activity of sole moringin by preparing different “tea” beverages from diverse MO sources at low and high temperatures, affecting myrosinase activity. The results showed an anti-inflammatory activity of the cold tea extract comparable with the one given by purified moringin, while the hot tea extract (containing just glucomoringin) had no effect on the suppression of inflammation. Jaafaru et al. [3] confirmed this finding with an *in vitro* model where cytotoxic and anti-proliferative processes on immortalized cells were triggered when treating with a moringin-rich extract from MO. The authors also performed in vivo studies for the evaluation of the toxic effects, not highlighted at high doses. More, MO leaf powder demonstrated hypoglycemic effects in diabetic subjects compared to healthy ones in a human clinical trial conducted in a Saharawi refugee camp [4].

A wider overview on the bioactive compounds present in *Brassicaceae* family plants, among which broccoli, kale, radish, and pack choi, has been provided in two consistent narrative reviews. The one by Jaafaru et al. [5] focused on the role of both glucosinolates and isothiocyanates, mainly sulforaphane, erucin, moringin, and phenethyl isothiocyanate, in preventing the risks associated with neurodegenerative disease development. Moreover, the review by Abellán et al. [6] described the preventive role in the development of several chronic diseases of plant sprouts, which could improve their phytochemical content with certain pre- and post-harvest conditions. These findings have also been supported by several research papers included in this Special Issue, with both in vitro and in vivo models. Concerning the in vitro studies, Alfarano et al. [7] considered the activity of sulforaphane, one of the isothyocianate products of glucoraphanin hydrolysis, on the glyoxalase 1 gene expression in human liver cancer models, and the relative activity of its transcript in the modulation of the intracellular antioxidant system. Another isothiocyanate with increasing interest for its bioactivity is sulforaphene, a product of glucoraphenin enzymatic hydrolysis, particularly abundant in radish. Kntayya et al. [8] demonstrated with immune-histochemical and molecular biology techniques the significant induction of apoptosis and cell cycle arrest in HepG2 cancer cells treated with sulforaphene. Concerning human studies, a German research group tested two beverages formulated with leaf powder of *Brassica carinata*, also called the Ethiopian kale—one rich in sinigrin and another one in its hydrolysis product, allyl isothiocyanate (AITC) [9,10]. Healthy subjects were enrolled in a randomized controlled cross-over study with a five-day consumption of the drinks and seven-day washout period [9]. After 2 h of consumption, higher amounts of AITC glutathione or cysteine conjugates were found in plasma after consumption of the AITC-rich beverage than the other drink, confirming the need of myrosinase for glucosinolate hydrolysis [9]. With regards to biological activity, contrasting results were found. In fact, the protection from macrophage DNA damage was significantly improved by the sinigrin-rich drink consumption compared to the AITC one, while the levels of pro-inflammatory prostaglandins were significantly lower in the plasma of the AITC-rich drink consumers than the sinigrin-rich ones [9]. The authors also found increased telomerase activity in lymphocytes upon consumption of the sinigrin-rich beverage compared to the AITC-rich drink [10]. On the contrary, the study of Fahey et al. [11] found significant results in terms of increased expression of genes, triggering inflammation, and antioxidant responses in healthy volunteers only when high amounts of isothiocyanates were found in the biological fluids of volunteers. To do this, a coated pill source of glucosinolates and myrosinase was developed, and the authors demonstrated that the capsule coating saved the myrosinase enzyme from its unfolding caused by the stomach acidity [11].

When taking into account the main group of phytochemicals in nature, (poly)phenols, notable advancements were published within this Special Issue, with up to eight studies. Three systematic reviews shed light on the role of (poly)phenols in the prevention of chronic diseases. Among these, Del Bo’ et al. [12], provided an overview of the last 10-year literature on the association of phenolic intake and specific disease markers and endpoints. Some subclasses of flavonoids were related to a low risk of diabetes, cardiovascular events, and all-cause mortality. Nevertheless, the authors acknowledged that there is huge heterogeneity in the available literature and that further studies should be conducted to better define specific recommendations related to (poly)phenol intake [12]. A second systematic review was conducted by Martini et al. [13] on the inter-individual variability in the cardiometabolic response to the consumption of hydroxycinnamic acids, as part of the COST Action FA1403 POSITIVe (Interindividual variation in response to consumption of plant food bioactives and determinants involved). This study highlighted how the effect of main dietary phenolic acids on cardiometabolic risk factors might be greater in individuals at higher cardiometabolic risk. In particular, higher baseline cholesterol, blood pressure, and glycemia are risk factors that might enhance the effectiveness of hydroxycinnamic acids, regardless of the food source [13]. A third systematic review and meta-analysis on the evidence of green tea consumption and risk of breast cancer and recurrence by Gianfredi et al. [14] pooled eight cohort studies and five case-control studies, to a total of 163,810 people. An inverse statistically significant relationship between green tea drinking and breast cancer risk was observed, showing a potential protective effect of green tea on breast cancer, especially its recurrence. Menopausal status conditioned the results of these observations, since green tea intake showed a significant protective role only in pre-menopausal women, with no effect in post-menopausal ones [14]. An epidemiological study also assessed the role of (poly)phenols and (poly)phenol-rich foods in the prevention of breast cancer. In detail, Wu et al. [15] evaluated whether long-term intake of whole-grain rye and whole-grain wheat, estimated using both a food frequency questionnaire (FFQ) and alkylresorcinol concentrations in adipose tissue biopsies, was related to the risk of developing invasive breast cancer in the Danish “Diet, Cancer, and Health” cohort. The consumption of both cereals was not associated with the risk of breast cancer for the whole population; however, after intake adjustment, women in the highest quartile of relative whole-grain rye intake exhibited a higher risk of overall and estrogen-receptor-positive breast cancer than women in the lowest quartile. The risk of estrogen receptor-negative breast cancer incidence was unaffected by rye consumption [15]. These results are of interest and deserve further research to understand the role of specific cereals in reducing or increasing breast cancer risk.

The remaining articles related to (poly)phenol research were focused on the food matrix, cell studies, or animal experiments. Iglesias-Carres et al. [16] investigated the optimized extraction of bioactive (poly)phenols from whole red grapes. This is of interest to further studying the bioactivity of the compounds in the extracts. Bianchi et al. [17] looked at the role of key dietary flavan-3-ols, (+)-catechin and procyanidin B_2_, in protecting the barrier function of intestinal cells undergoing inflammatory stress. The authors highlighted how, under the physiological conditions adopted, these compounds did not prevent inflammation-dependent impairment of the epithelial barrier function. Nevertheless, these flavan-3-ols modified the expression of tight-junctional proteins and, in particular, claudin-7, shedding light on the potential biological activity of these major dietary flavan-3-ols at the intestinal level [17]. Our research group, in collaboration with other teams, also assessed the capability of the main colonic metabolites of flavan-3-ols, phenyl-γ-valerolactones, to reach the brain by crossing the blood-brain barrier [18]. Up to five models, with increased level of experimental realism (including in silico, in vitro, and in vivo animal studies), were used to check this key aspect related to the bioactivity of flavan-3-ol at the neurological level. The results showed the blood-brain barrier permeability of one of the main flavan-3-ol microbial metabolites, a sulfate form of 5-(3’,4’-dihydroxyphenyl)-γ-valerolactone, supporting the hypothesis that the colonic metabolites of flavan-3-ol may be present in brain tissues. Last, another work assessed the prospects of phenolic bioactives at the brain level; Mastinu et al. [19] demonstrated that γ-oryzanol, a ferulic acid ester of phytosterols, was able to improve the cognitive performance at pharmacological doses in mice during the neuroinflammatory response to lipopolysaccharide through anti-inflammatory processes. 

Other sources of multiple classes of bioactive compounds were assessed in the contributions of this Special Issue. The work of Odongo et al. [20] focused on the characterization of the extract of the African nightshade (*Solanum scabrum*), a plant particularly rich in chlorogenic acids, flavonoids, carotenoids, and chlorophyll, and on the evaluation of its activity in the protection from DNA damage. The results showed that, among the different technological treatments, cooking and fermentation enhanced the extract protection from DNA damage and the induction of ARE/Nrf2- mediated gene expression [20]. Another interesting study was performed by Geddo et al. [21] on the ability of a fluid extract of black pepper (*Piper nigrum*), standardized in the sesquiterpene *trans*-β-caryophyllene, to reduce lipid accumulation in 3T3-L1 preadipocytes and improve glucose uptake in C2C12 myotubes. Amor et al. [22] also evaluated the potential benefits at the cardiometabolic level of an aged black garlic extract, rich in *S*-allyl cysteine and melanoidins, in male rats. On the other hand, an intervention study assessed the effect of consuming an acute dose of sofrito on the inflammatory status [23]. Sofrito is a Mediterranean tomato-based sauce that typically contains olive oil, onion, and garlic, with the bioaccessibility of carotenoids and (poly)phenols enhanced by the cooking process. This work by Hurtado-Barroso et al. [23] reported a significant decrease in C-reactive protein and tumor necrosis factor-α, the latter being inversely correlated with an increase in the urinary excretion of (poly)phenols and plasma β-carotene (but not lycopene) [23]. Of note, the authors pointed to the food matrix and not to a single compound or class of compounds to explain the biological effect observed.

In summary, several bioactives and aspects have been taken into account within this Special Issue. Obviously, much work is needed to better understand the metabolism, bioavailability, and biological properties of plant bioactives and solve current critical gaps. We hope these studies will inspire the readers to explore the frontiers of this research field.
